# Illuminating the Future of Catecholamine Detection

**DOI:** 10.1111/jnc.70535

**Published:** 2026-08-09

**Authors:** Julia Ackermann, Andreas Reiner, Sebastian Kruss

**Affiliations:** ^1^ Fraunhofer Institute for Microelectronic Circuits and Systems Duisburg Germany; ^2^ Department of Biology and Biotechnology Ruhr University Bochum Bochum Germany; ^3^ Department of Chemistry and Biochemistry Ruhr University Bochum Bochum Germany

**Keywords:** carbon nanotubes, catecholamines, fluorescence sensors, genetically encoded sensors, molecular neuroscience, near infrared

## Abstract

Catecholamines such as dopamine (DA) and norepinephrine (NE) are critical neuromodulators which influence a wide range of physiological and behavioral processes. Optical sensors/probes are powerful tools to detect catecholamines with high spatial and temporal resolution, enabling real‐time imaging in complex biological environments. In this review, we highlight recent advances in single‐walled carbon nanotube (SWCNT) sensors and genetically encoded sensors for fluorescence‐based catecholamine detection. We illustrate how these technologies have enabled new biological discoveries by allowing the spatiotemporal mapping of catecholamine release dynamics with unprecedented resolution. We discuss the complementary features of these techniques and remaining key challenges including molecular selectivity, tailored kinetics, and enhanced tissue penetration. Finally, we outline future directions and opportunities for integrating these technologies into neuroscience research, aiming to expand our understanding of catecholaminergic signaling across scales.

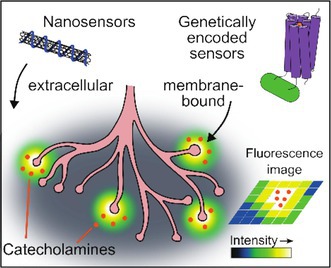

## Graphical abstract

Catecholamines such as dopamine and norepinephrine are key signaling molecules in our body. We discuss how fluorescent sensors (carbon nanotube based and genetically encoded) for these molecules in combination with novel imaging methods and kinetic data analysis enable new biological experiments.

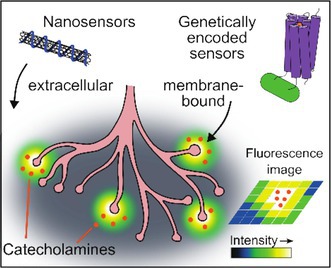

**Abbreviations**
2p: two‐photon
AAV: adeno‐associated virus
AR: adrenergic receptor
BSA: bovine serum albumin
CNS: central nervous system
cp‐FP: circularly permuted fluorescent protein
DA: dopamine
Em: emission
EPI: epinephrine
Ex: excitation
FFN: false fluorescent neurotransmitter
FLIM: fluorescence lifetime imaging
FP: fluorescent protein
GFP: green‐fluorescent protein
GPCR: G protein‐coupled receptor
ICL3: third intracellular loop
NE: norepinephrine
NIR: near infrared
ssDNA: single‐stranded DNA
SWCNT: single‐walled carbon nanotube

## 1 Introduction

Catecholamines such as dopamine (DA), norepinephrine (NE, noradrenaline) and epinephrine (EPI, adrenaline) act as hormones and neuromodulators with a wide range of modulatory actions for controlling body as well as brain functions (Kandel et al. 2021). DA contributes to movement control and motivation/reward systems. NE increases alertness and attention, as well as heart rate and blood pressure. EPI further acts as hormone and prepares the body for fight‐or‐flight responses. Consequently, catecholamines play also major roles in neuropsychiatric and neurological diseases, including Parkinson's disease (DA), depression, anxiety, schizophrenia and addiction.

In the central nervous system (CNS) catecholamines are produced by a rather small set of neurons localized in defined nuclei, for example, in the substantia nigra, the ventral tegmental area and hypothalamus for the case of DA (Cragg and Walton 2025), and the locus coeruleus for NE (Ordway et al. 2007). Nevertheless, the projections of these catecholaminergic neurons reach throughout the midbrain, most cortical regions and the cerebellum thereby providing coordinated modulation across different brain regions.

The last decades brought great insights by identifying catecholamine‐producing neurons and mapping their projections, which typically relied on using their biosynthetic enzymes as markers, for example, by staining for tyrosine hydroxylase, which catalyzes the first step of catecholamine biosynthesis. Similarly, great progress has been made in identifying the corresponding DA and adrenergic receptors (ARs) that decode these signals along with their pharmacology, which led to numerous approved medical treatments (Beaulieu and Gainetdinov 2011). However, despite these achievements, many details remain unknown. This includes fundamental questions such as how, where, when and how many of these modulators are released and on which spatial scales these signals travel in tissues (Liu and Kaeser 2019).

Moreover, there is increasing evidence that catecholamines play also an important role in the immune system. It is known that certain T‐cells use catecholamines for signaling and overall many parts of the catecholamine machinery known from the brain are also present in cells outside the CNS (Maceiras et al. 2017; Bergquist et al. 1994; Flierl et al. 2007; Papa et al. 2017).

**FIGURE 1:**
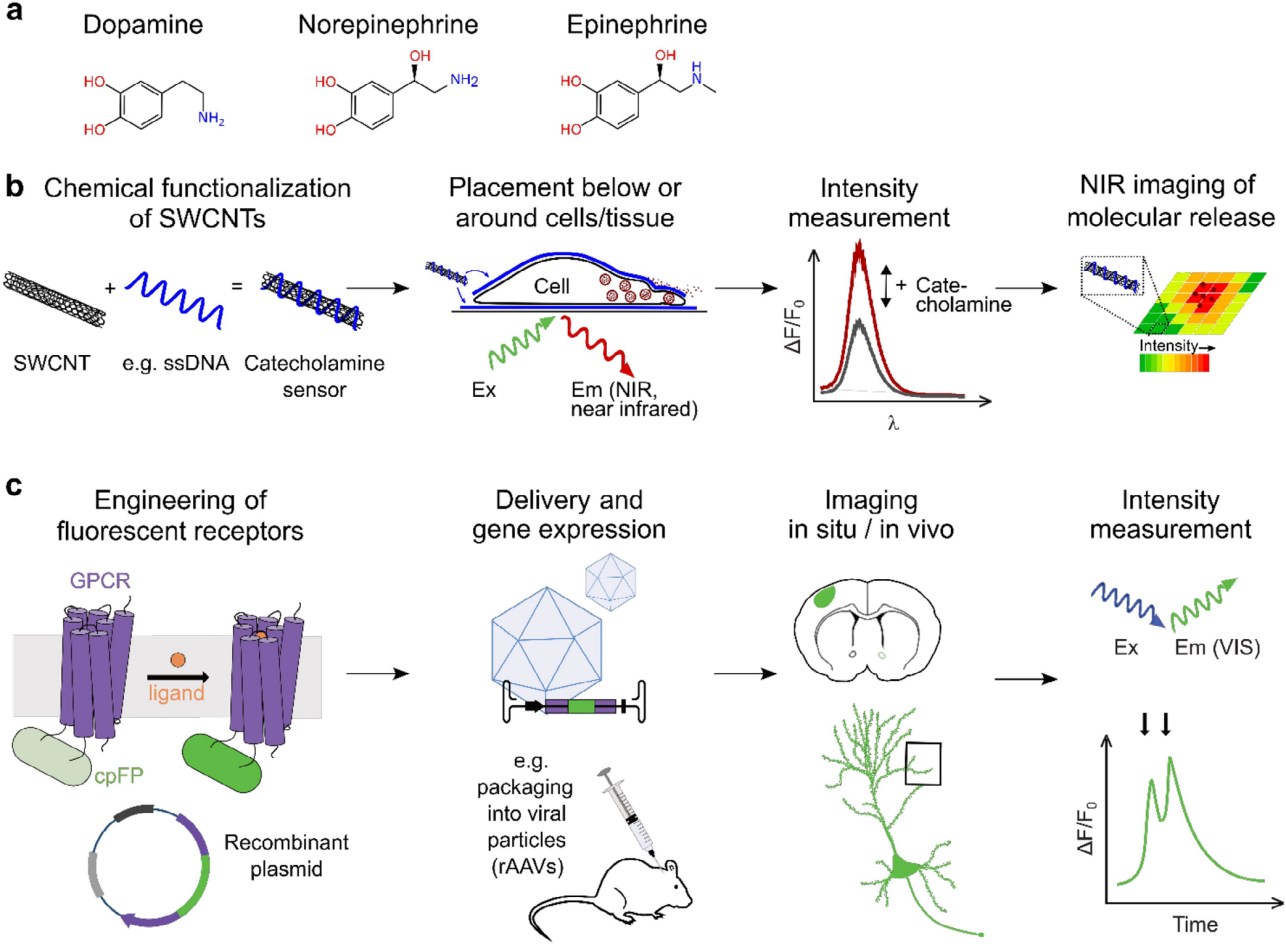
Strategies and process flow for optical catecholamine detection. (a) The three main catecholamines. (b) NIR fluorescent SWCNTs are rendered sensitive to catecholamines through functionalization (e.g., with ssDNA). These sensors are placed below or on top of cells or in tissue. They emit NIR fluorescence (830–2400 nm) that changes upon interaction with catecholamines. (c) Genetically encoded fluorescent sensors can be engineered by inserting a cp‐FP, for example, cp‐EGFP, into a suitable GPCR backbone. The corresponding genes are introduced into cells or animals, for example, by transduction with viruses. Neurotransmitter dynamics can then be visualized in certain brain regions or cell types, even in living animals.

Research in this area has for the largest part been hampered by a lack of suitable techniques to detect catecholamines in live tissues and complex chemical environments with high specificity and spatiotemporal resolution (Zheng and Li 2023). This is also due to the high structural similarity of the catecholamines (Figure 1a), which complicates their selective differentiation. Microdialysis combined with analytical methods such as liquid chromatography allows selective quantification of extracellular catecholamines in vivo, but it is invasive, offers limited spatial resolution, and is restricted to minute‐scale sampling, making it unsuitable for fast dynamics (Wu et al. 2022; Post and Sulzer 2021). Electrochemical approaches such as amperometry and voltammetry offer high temporal resolution and are less invasive than microdialysis, yet their spatial resolution remains constrained by the number, type, and placement of electrodes (Wu et al. 2022; Post and Sulzer 2021). Optical methods, in contrast, typically provide higher spatial resolution and reduced invasiveness. However, methods such as false fluorescent neurotransmitters (FFNs) or vesicle‐loading dyes report on exocytosis only indirectly by marking cellular components and are limited by issues such as photobleaching (Wu et al. 2022; Post and Sulzer 2021). Related small‐molecule fluorescent probes have also been developed to monitor catecholamine release more directly. For instance, high‐affinity probes capable of selectively detecting NE over DA have enabled imaging of NE exocytosis at the single‐vesicle level (Zhang et al. 2019).

In this overview article, we review and discuss in our opinion most promising techniques in terms of spatiotemporal resolution and selectivity.

## 2 Optical Strategies for Catecholamine Imaging

### 2.1 SWCNT‐Based Catecholamine Sensors

Semiconducting single‐walled carbon nanotubes (SWCNTs) offer a powerful platform for optical mapping of catecholamine release. With diameters of 0.4–3 nm (Baughman et al. 2002) and lengths of around 600 nm after functionalization (Nißler et al. 2019), these one‐dimensional, hollow nanostructures exhibit chirality‐dependent narrow‐band fluorescence in the near infrared (NIR, 830–2400 nm) range (Bachilo et al. 2002). Unlike conventional fluorophores, SWCNTs are exceptionally photostable, exhibiting no blinking or photobleaching, which makes them well‐suited for long‐term imaging (Godin et al. 2017). Their emission falls within the NIR biological transparency window, in which reduced light scattering and minimal tissue autofluorescence result in improved signal‐to‐noise ratios (Hong et al. 2017). NIR emission also offers the advantage of being compatible with many other optical methods, such as dye staining to obtain additional structural information on specific cellular compartments, without spectral interference. This leads to ideal conditions for imaging in complex biological environments. In addition, SWCNTs are highly sensitive to their chemical surroundings due to their one‐dimensional structure and all‐surface character. Changes in the local environment can change the SWCNT fluorescence, which makes them nanoscale sensors (Ackermann et al. 2022). Through surface functionalization, their molecular recognition can be tailored to specific analytes, including catecholamines such as DA or EPI (Kruss et al. 2014).

Most current catecholamine sensors are based on the adsorption of single‐stranded DNA (ssDNA) onto the SWCNT surface, forming a so‐called corona phase. Appropriate recognition layers are selected through a corona‐phase molecular recognition (CoPhMoRe) screening, where different ssDNA sequences are tested for their selective interaction with target analytes. Both sensor sensitivity and selectivity depend on the exact ssDNA sequence (Mann et al. 2017). Catecholamine binding typically results in increased fluorescence intensity. This response arises from analyte‐induced conformational changes of the ssDNA that alter the environment around the SWCNT (Kruss et al. 2017). Moreover, analytes like DA appear to influence the coupling between the ssDNA‐SWCNT complex and its hydration shell, which changes the fluorescence quantum yield (Nalige et al. 2024). Recent experimental and computational studies further suggest that the fluorescence response in ssDNA‐SWCNT catecholamine sensors correlates positively with the electron density on the aryl motif of the analyte (Krasley et al. 2025). SWCNT‐based sensors have a sensitivity down to the single molecule level and typical *K*_d_ values in the low μM range which can be tuned by the DNA sequence (Gretz et al. 2025).

In addition to ssDNA‐based designs, SWCNTs functionalized with boronic acid quantum defects have been developed. These constructs enable ratiometric sensing via one stable reference fluorescence emission peak (~990 nm) and one quantum defect‐related emission peak (~1130 nm) that decreases upon catecholamine binding (Ma, Mohr, et al. 2024).

For use in cellular systems, the sensors can either be applied directly on the cells or tissues (Elizarova et al. 2022; Sistemich et al. 2023) or used to coat substrates prior to cell seeding (Kruss et al. 2017; Ackermann, Stegemann, et al. 2023). The first approach reduces prolonged exposure of sensitive cells to SWCNTs (even though SWCNTs are highly biocompatible (Nadeem et al. 2023)) and simultaneously minimizes nonspecific protein adsorption onto SWCNTs or potential contamination with bacteria onto the SWCNTs during culture (Elizarova et al. 2022). The second approach ensures a homogeneous sensor distribution across the entire substrate, allowing reliable and reproducible detection of molecular release events (Ackermann, Reger, et al. 2023).

Changes in fluorescence can be interpreted as localized extracellular catecholamine release events, effectively turning SWCNTs into a chemically sensitive “sensor” pixel (Figure 1b). This enables spatiotemporal mapping of neurotransmitter dynamics with up to ~100 ms in time and hundreds of nanometers precision in space (Kruss et al. 2017). These capabilities have provided biological insights that would be difficult or impossible to obtain using classical catecholamine detection techniques and will be discussed in detail below.

### 2.2 Genetically Encoded Catecholamine Sensors

Another powerful approach for bioanalyte detection is to utilize naturally occurring binding proteins and to engineer them into fluorescent biosensors. In vivo, catecholamines are primarily detected by G protein‐coupled receptors (GPCRs), namely DA receptors and ARs. Receptors from both families have been successfully converted into catecholamine sensors by inserting circularly‐permuted versions of green‐fluorescent protein (cp‐GFP) (Baird et al. 1999) or related circularly permuted fluorescent protein (cp‐FP) modules into their third intracellular loop (ICL3) (Figure 1). Analyte binding at the extracellular binding site triggers conformational changes in the GPCR scaffold mostly involving transmembrane helices TM5 and TM6, which then cause a fluorescence intensity increase or decrease of the cp‐FP module. Despite their rather modular design and the successful integration of cp‐GFP modules into many biosensors such as GCaMPs (Zhang and Looger 2024), the development of well‐performing sensors typically requires optimization. This typically involves screening of several hundred to thousand sensor variants with alterations in their linker regions and other amino acid side chains for improved expression levels, sensor brightness, large signal changes, photostability, affinity or other desirable properties. No single sensor will serve all purposes, because of inherent trade‐offs, for example, between affinity, kinetics and temporal resolution. However, having a palette of sensors with different properties, allows to choose a sensor with characteristics that are most needed for a particular application and biological question. For genetically encoded sensors the production of them in the cells might affect their biology, they will occupy membrane space and they will bind free catecholamines and affect their extracellular concentration.

The first GPCR‐based sensors for catecholamines were built on the human DA receptors DRD1 and DRD2 and called dLight sensors (Patriarchi et al. 2018) and GRAB_DA_ sensors (Sun et al. 2018), respectively. These sensors inherit key properties from their parent receptors, for example, DRD2‐based sensors have a high DA affinity in the nanomolar range, whereas DRD1‐based sensors only show saturation in the micromolar range; both receptor types provide an at least 10‐fold higher affinity for DA than NE and EPI (Patriarchi et al. 2018; Sun et al. 2018; Klein Herenbrink et al. 2022). Using a classical cpGFP module (Baird et al. 1999) also present in GCaMP and optimizing its insertion, reversible fluorescence increases up to 9‐fold over baseline with subsecond response kinetics were obtained with dLight1.2 (Patriarchi et al. 2018), whereas higher affinity GRAB_DA_ sensors exhibit slower OFF kinetics on the seconds timescale (Sun et al. 2018). Placing the cpGFP module within ICL3 proved quite useful for detecting conformational changes, but it also disrupts the coupling to G proteins and activity‐dependent receptor internalization. This ensures minimal interference with native signaling, which makes these sensors well tolerated for in vivo applications and enables stable, long‐term monitoring. Still, the expression of sensor proteins may elicit indirect biological perturbations, for example, by affecting protein trafficking or the composition of signaling domains in the corresponding cells. Long‐term buffering of analytes could cause adaptation for example in terms of the amount of catecholamine release.

Both, the original dLight and GRAB_DA_ sensors, have been subsequently optimized and modified (Patriarchi et al. 2020; Sun et al. 2020; Nakamoto et al. 2021; Zhuo et al. 2024). Notably, replacing the cpGFP module with cpmApple, provided access to red‐fluorescent sensor variants with emission maxima of ~590 nm. The specifics of these DA receptor‐based sensors have been summarized in two recent reviews (Labouesse et al. 2020; Dong et al. 2025). A newer version, dLight3.6, brings again higher affinities and is thus well suited for probing release in brain regions that receive only sparse dopaminergic innervation (Tian et al. 2025).

Similarly, detection of NE has been achieved by relying on ARs as sensor scaffold. This started with different affinity GRAB_NE_ sensors that were based on an *α*2AR scaffold and have now been improved further (Feng et al. 2019, 2024). In parallel, this toolset was extended with green‐ and red‐fluorescent *α*1AR‐based sensors, nLightG and nLightR, respectively (Kagiampaki et al. 2023; Rohner, Curreli, et al. 2026). These NE sensors provide reasonable specificity for NE over DA, but, like their physiological counterparts (here *α*2AR), they also bind EPI with similar affinity and response magnitude (Feng et al. 2024). However, in the CNS EPI is only found in very low amounts and specialized functions in contrast to its widespread role as body hormone. Recently also a high‐affinity NE sensor was developed based on the α1D‐AR scaffold (Rohner, Kagiampaki, et al. 2026).

Since these sensors are protein‐based they can be genetically encoded, that is, they can be expressed in any cell by introducing the respective coding DNA sequences. The preferred way of delivery depends on the specimen. For transient expression of cultured cells different transfection protocols are available, whereas most in situ and in vivo applications rely on viral transductions, for example, DNA packaging into recombinant adeno‐associated virus (AAV) particles. These techniques are now established in many neuroscience labs and enable expression that can last from days to months. A key advantage of these genetic approaches is that sensor expression can be limited to specific cell types by using suitable genetic driver or control elements, for example, by using neuron‐ or astrocyte‐specific promoters or utilizing animals that express Cre‐recombinase in a cell‐type specific manner. By injecting AAVs into specific brain areas, sensor expression can be further directed to selected brain areas and to distinct projections originating from them, for example, from the locus coeruleus to the hippocampus (Rohner, Curreli, et al. 2026). Due to this specificity in sensor expression, specific catecholamine signals can also be recorded using fiber photometry‐based approaches, which gives access to relevant brain structures without the need for deep tissue imaging. Given their in vivo applicability (also reviewed in (Lin et al. 2021; Zhong et al. 2025; Labouesse et al. 2020; Dong et al. 2025)), genetically encoded catecholamine sensors were rapidly adapted by the neuroscience community, as will be illustrated in the next chapter.

## 3 Application and Novel Insights

SWCNT‐based sensors and genetically encoded sensors both enable catecholamine imaging with high spatial and temporal precision that exceeds conventional methods. Their respective strengths, like extracellular sensitivity and high photostability for SWCNT sensors, or cell‐type specificity and subcellular targeting for genetically encoded sensors, make them powerful tools for addressing diverse biological questions. The following examples illustrate how these approaches already have provided new insights into catecholamine signaling.

**FIGURE 2:**
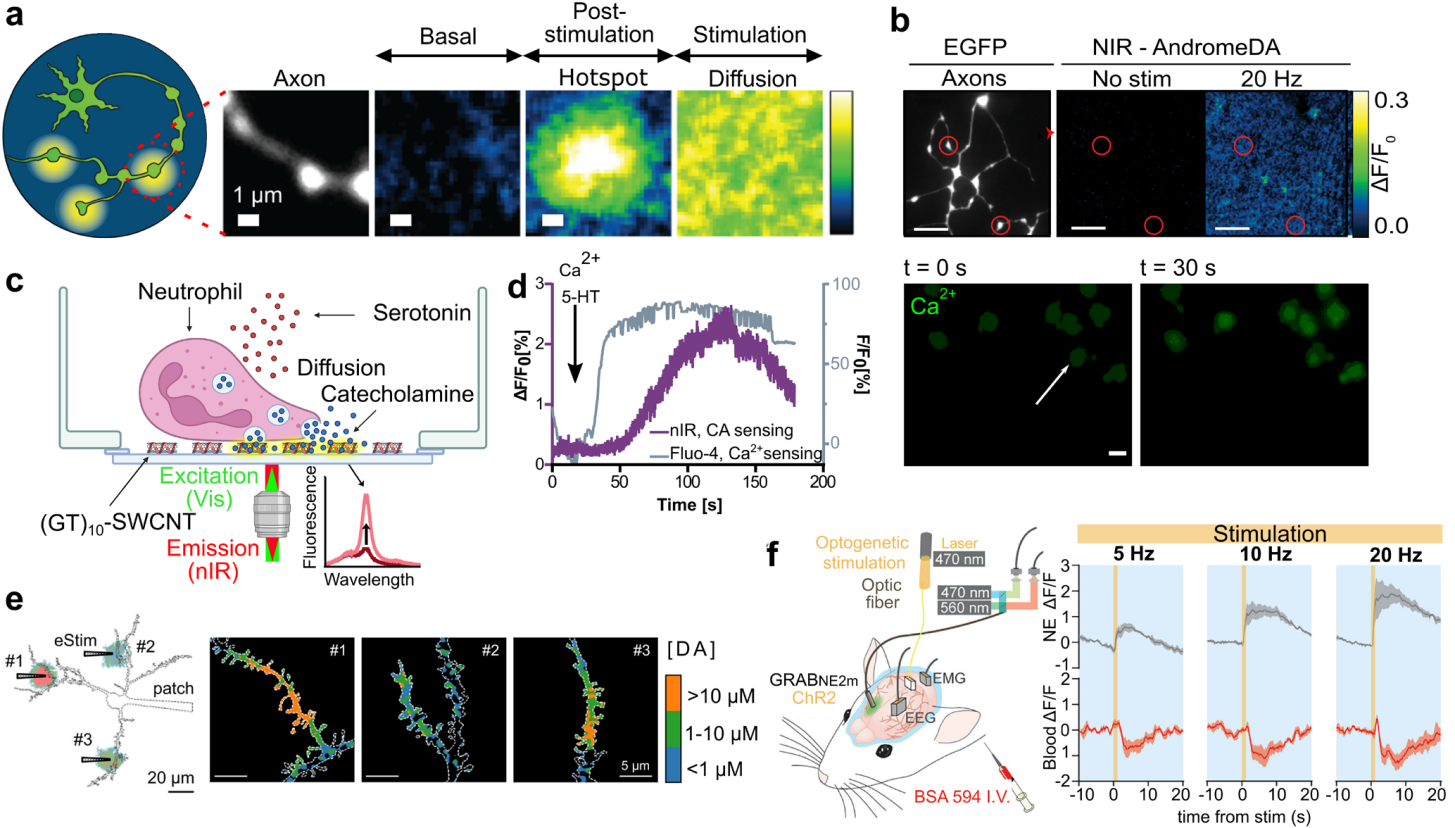
Insights obtained using optical catecholamine sensors. (a, b) SWCNT sensors are used as paint to visualize the spatiotemporal dynamics of the formation of DA release hotspots and extracellular diffusion from dopaminergic neurons. Adapted from Ref. (Elizarova et al. 2022) with permission. (c) Serotonin triggers intracellular Ca^2+^ signals, followed by exocytosis of catecholamines from human immune cells (neutrophils). (d) These releases can be visualized in parallel using a Ca^2+^ indicator (Fluo‐4 AM, gray) and a fluorescent SWCNT coating (purple). The images on the right show the outlines of the cells stained with the Ca^2+^ dye. Adapted from Ref. (Mohr et al. 2025). (e) DA imaging with dLight1.3b (2p imaging) in a brain slice shows locally restricted and heterogeneous DA release upon local electrical stimulations in different regions (#1–3). Reprinted with permission from Ref. (Yee et al. 2025). (f) In vivo fiber photometry measurements of GRAB_Ne2m_ in the dorsal cortex show evoked NE release (dF/F increase) after optogenetic stimulation of neurons in the locus coeruleus using channelrhodopsin. The NE release acts to reduce blood flow (detected with bovine serum albumin (BSA)‐Alexa Fluor 594). Adapted from Ref. (Hauglund et al. 2025), Copyright 2024, with permission from Elsevier.

SWCNT‐based sensors have enabled direct visualization of catecholamine dynamics across various biological systems, ranging from pheochromocytoma (PC12) cells (Kruss et al. 2017) to primary neurons (Elizarova et al. 2022; Bulumulla et al. 2022) to brain slices (Beyene et al. 2019). A particular strength of SWCNT‐based sensors is that, by coating an entire surface or adsorbing (“painting”) the sensors onto cells, release processes can be monitored over a wide area simultaneously. This capability was first fully exploited by Elizarova et al. using the AndromeDA approach (Elizarova et al. 2022). Primary dopaminergic neurons were cultured, and DA release was simultaneously detected from up to 100 axonal varicosities. Because the neurons expressed enhanced green‐fluorescent protein (EGFP), the localized hotspots of release could also be directly correlated with subcellular structures (Figure 2a,b). The study revealed that release hotspots are highly heterogeneous, occurring at only about 17% of all varicosities, indicating that many release sites are functionally silent. Moreover, DA release at these hotspots was found to require Munc13, demonstrating that AndromeDA can also be used for studying the molecular mechanisms underlying DA secretion.

In a further study, Bulumulla et al. employed SWCNT‐based nanosensors to investigate DA release from dendritic processes of dopaminergic neurons, a phenomenon that has remained poorly understood (Bulumulla et al. 2022). Release occurred as hotspots with a mean spatial spread of about 3.2 μm and a frequency of roughly one hotspot every 7.5 μm of dendritic length. These dendritic events were shown to be fast and Ca^2+^‐dependent, indicating a release mechanism reminiscent of classical active zones. Retrospective super‐resolution imaging combined with immunostaining further revealed enrichment of the presynaptic scaffolding protein Bassoon at dendritic release sites, suggesting that dendritic DA release is organized through an active zone‐like molecular machinery. More recently, this nanosensor approach has been extended to animal models, demonstrating its applicability for monitoring dopamine signaling in living systems (Mun et al. 2026).

More recently, Mohr et al. extended the use of SWCNT sensors to the immune system by studying catecholamine release from human neutrophils (Mohr et al. 2025). While previous studies had suggested a role for catecholamines in neutrophils, the underlying mechanism remained unclear. It was shown that neutrophils synthesize, store, and release catecholamines using machinery analogous to that in neurons. This was demonstrated through a combination of different methods, including immunostaining of key components of catecholamine metabolism, but especially corroborated through SWCNT‐based extracellular imaging of catecholamine release. Furthermore, it was found that serotonin released by activated platelets triggers Ca^2^^+^‐dependent exocytosis of catecholamines (Figure 2c,d). This paracrine feedback loop between neutrophils and platelets affects certain immune functions such as NETosis and platelet aggregation and demonstrates that catecholamine signaling extends beyond the nervous system to directly regulate immune functions. From a more technical perspective, this demonstrates that terminally differentiated and short‐lived cells such as neutrophils can be studied in this manner, as they cannot be readily transfected with genetically encoded sensors. Moreover, the study highlights that detecting transient and local catecholamine release is crucial.

Genetically encoded sensors allow to probe local catecholamine release and to differentiate between tonic (basal) and phasic (activity‐dependent) release mechanisms. A remarkable, new finding was obtained by Liu et al. (2022), who expressed GRAB_DA2m_ in dopaminergic neurons and, by performing microscopy on acute brain slices, detected spontaneous DA release events in their striatal axons, which were apparently triggered by ectopic, that is, non‐somatic action potentials. The authors further showed that these locally produced DA signals may be triggered by cholinergic activation of nicotinic acetylcholine receptors (nAChRs), which are located on the axons of the dopaminergic neurons. More lately, an in vivo study has suggested that this mechanism might serve to limit axonal release (Zhang et al. 2025). Similarly, dLight1.3b measurements revealed discrete and sparse subcellular DA release on dendrites triggering local receptor responses (Figure 2e) (Yee et al. 2025). Also, for NE, here using GRAB_NE2m_, a strong axonal modulation of the release has been observed (Ma, Li, et al. 2024). Using two‐photon (2p) imaging in the prefrontal cortex of mice, it was found that increased adenosine levels resulting from microglial activity lowered extracellular NE levels, which appeared to be responsible for the observed sleep‐promoting effects.

Besides investigating cellular and subcellular release properties, genetically encoded sensors are also particularly well‐suited for visualizing neuromodulatory circuit functions. For instance, using GRAB_NE_ sensors, slow NE oscillations have been observed and linked to sleep regulation (Osorio‐Forero et al. 2025). There NE, which is a potent vasoconstrictor too, drives slow vasomotion thereby enabling glymphatic clearance during sleep (Hauglund et al. 2025). In this study the authors also made use of optogenetic stimulation of NE‐releasing neurons in the locus coeruleus (using channelrhodopsin), while in parallel recording the resulting NE level increases in the cortex using fiber photometry (Figure 2f). Further physiological insight into the action of catecholaminergic neurotransmitters was also obtained in the study of Hasegawa et al. (2022). Here, fiber photometric measurements of GRAB_DA_ were used to compare how the DA levels changed during sleep transitions in different brain regions. By correlating various processes, the authors could show that the DA release in the basolateral amygdala appears to be a trigger for rapid eye movement (REM) sleep.

Apart from mice, genetically encoded sensors have also found use in other animals. For instance, dLight fiber photometry measurements were recently performed in monkeys to study the contribution of DA to stimulus–reward associations in the striatum of primates (Yan et al. 2025). In another recent study GRAB_NE2h_ was used to show that ketamine caused elevated the NE levels in the hindbrain of zebrafish larvae with profound behavioral effects (Duque et al. 2025).

## 4 Key Challenges and Future Prospects

**FIGURE 3:**
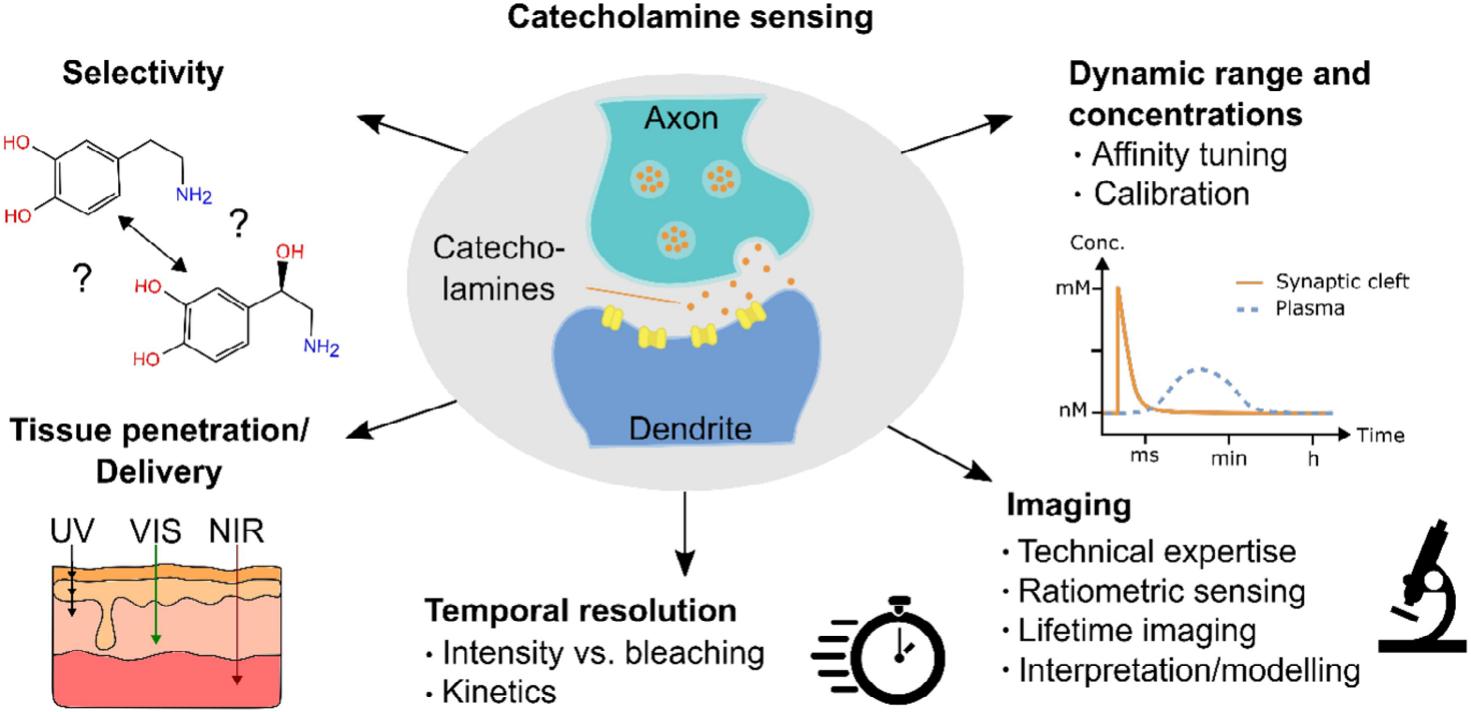
Key challenges in optical catecholamine sensing. Selectivity refers to the difficulty of distinguishing structurally similar neurotransmitters, especially in complex biological environments. Tissue penetration reflects the limitations of artifact‐free optical signal detection in deep brain regions and the efficient delivery of sensors to specific biological structures. Temporal resolution highlights the need to capture rapid neurotransmitter dynamics. Dynamic range refers to the need for sensitivity, that is, affinity‐tailored sensors across distinct biological contexts and their calibration. Imaging refers to specific technical expertise and know‐how for data analysis, especially for advanced approaches such as ratiometric sensing and lifetime imaging.

The first genetically encoded sensors such as Ca^2+^‐detecting GCaMPs have been established decades ago, and with the application to GPCRs they now have another transformative impact on neuroscience. Their successful deployment highlights how optical tools can fundamentally reshape our understanding of signaling processes in living systems. Similarly, SWCNT‐based sensors have recently opened new opportunities for mapping extracellular catecholamine dynamics with high spatiotemporal resolution. Yet, despite these achievements, both approaches face important challenges that need to be addressed to enable their broader use in complex biological environments and signaling paradigms (Figure 3).

### 4.1 Selectivity

One challenge that exists for almost all types of sensors in catecholamine detection is selectivity. Due to their chemically similar structure, cross‐sensitivities often occur. However, even in nature the specificity of receptors for catecholamines is not absolute. To increase the selectivity of SWCNT‐based sensors, Ma et al. demonstrated that combining boronic acid quantum defects with a second functionalization chemistry, such as charged polyethylene glycol polymers, can enhance catecholamine recognition (Ma, Mohr, et al. 2024). Furthermore, a rational approach based on functionalization with aptamers in combination with passivation agents recently demonstrated improved sensitivity and selectivity with respect to interfering molecules (Stefoni et al. 2026). Another promising route toward improved selectivity, applicable to both SWCNTs and genetically encoded sensors, is high‐throughput screening of sensor variants (e.g., modifications in linker regions or binding pockets) directly under biologically relevant conditions.

### 4.2 Tissue Penetration/Delivery

Beyond selectivity and sensor robustness, the applicability of optical sensors in vivo is strongly influenced by tissue penetration, which depends on how effectively light can be delivered and detected in scattering tissue. Because scattering and autofluorescence decrease toward the NIR region, signal‐to‐noise ratios improve and penetration depth increases (Hong et al. 2017). In this regard, SWCNT‐based sensors are intrinsically well positioned, as they emit above 880 nm and can either be excited directly in the NIR or benefit from large Stokes shifts (~300 nm), minimizing spectral crosstalk. Most genetically encoded catecholamine sensors, however, are currently restricted to the green and red spectral range, where background signals, including hemodynamic signals, are more pronounced (Wu et al. 2022). Extending their emission into the far‐red and NIR has proven difficult, as fluorescent proteins in this range are generally less bright and apparently more challenging to engineer into circularly permuted variants, which are required for integration into GPCR scaffolds (Shcherbakova 2021). To overcome these limitations, a recent study replaced the cpFP module of a DRD1‐based sensor with a circularly permuted HaloTag (Deo et al. 2021), which allows installation of synthetic fluorophores, that is, rhodamine dyes to obtain emission > 650 nm (Zheng et al. 2025). This approach extends the accessible spectral range into the far‐red/NIR region and offers higher brightness, improved photostability, and potentially greater penetration depth for in vivo applications. New wavelength regimes also allow for better multiplexing of multiple sensors and/or optogenetic actuators, such as channelrhodopsins.

Beyond optical accessibility, delivery (i.e., biological targeting) of the sensor represents another important challenge for in vivo applications. Genetically encoded sensors require genetic manipulation, typically via transfection into cultured cells or viral transduction in tissues and organisms, which introduces additional biological and technical complexity. However, this strategy enables cell‐type‐specific targeting and stable long‐term expression. In contrast, SWCNT‐based sensors do not require genetic modification but must be delivered exogenously to the tissue of interest. Delivery by local injection followed by diffusion through the extracellular space is feasible (Godin et al. 2017), but precise targeting to defined cell types or anatomical regions remains to be addressed. For extended applications of hours and days also biocompatibility, for example, toxicity and stability (turn‐over), become additionally relevant aspects.

### 4.3 Temporal Resolution

While the spectral properties determine how well signals can be detected in deep tissue, temporal resolution determines whether the rapid dynamics of neurotransmitter release can be accurately recorded. Many neurotransmitter events occur on the millisecond‐to‐second timescale and are highly diffusive, so resolving them in real time requires both sufficient signal intensity and rapid sensor responses. Higher fluorescence intensity allows shorter camera exposure times and thus higher frame rates. For genetically encoded sensors, signals can be enhanced by increasing excitation intensity, though this comes at the cost of photobleaching. Sensors with high signal changes due to ground‐state excitation changes and high fluorescence quantum yields are advantageous in this context. SWCNT‐based sensors, in contrast, are exceptionally photostable, but their quantum efficiency is comparatively low. Strategies such as the introduction of quantum defects, have been shown to increase the quantum yield and could improve overall brightness (Brozena et al. 2019).

**FIGURE 4:**
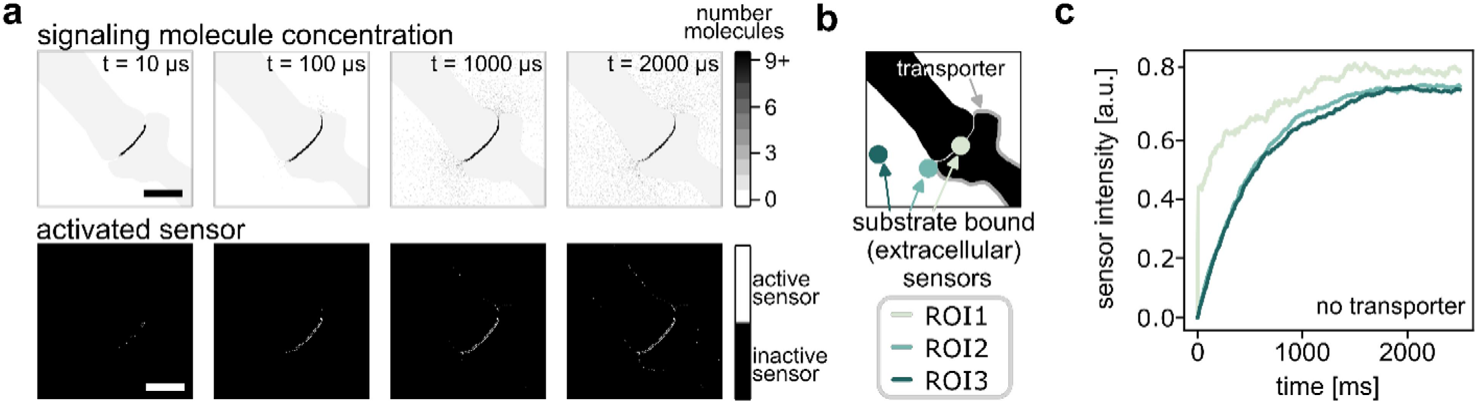
Simulation of spatial and temporal dynamics of DA binding in and around a synapse. (a) Spatiotemporal evolution of DA concentration (top) and the corresponding distribution of activated receptors of an idealized membrane‐bound sensor (bottom); (b) Position of three sensors located at increasing distances from the release site. (c) Time course of the sensor intensity detected by the three sensors in the absence of DA transporters. Reproduced with permission from Ref. (Gretz et al. 2026).

Beyond signal intensity, temporal resolution is fundamentally constrained by the binding kinetics of the sensor. The association and dissociation rates determine how rapidly a sensor responds to concentration changes and thus which processes can be resolved. If the sensor kinetics are slower than the underlying catecholamine dynamics, the measured fluorescence signal does not directly reflect the current state (concentration). In fact, the fluorescence intensities reflect the number of occupied binding sites rather than the actual analyte concentration. A further factor influencing the measured signal is the distance between the sensor and the release site. For membrane‐bound sensors, such as genetically encoded sensors located at cell membranes, the signal response may differ from that of SWCNT‐based sensors, which might detect catecholamine molecules at greater distances within the extracellular space. This results in a different sensor occupancy on the time axis (Figure 4) (Gretz et al. 2026). Knowledge of the kinetic parameters is therefore essential for interpreting concentration dynamics in non‐equilibrium systems, so that the actual state can be derived by backward simulation. Kinetic models can therefore establish a link between the measured fluorescence signals and the underlying neurotransmitter dynamics/concentrations.

To address this, Meyer et al. developed a theoretical framework simulating the fluorescence response of sensors (arrays) to concentration gradients (Meyer et al. 2017). Their theoretical analysis suggested that accurate mapping of neurotransmitter release requires rate constants on the order of *k*_on_ = 10^6^ M^−1^ s^−1^ and *k*_off_ = 10^2^ s^−1^, corresponding to a high *K*_d_ value of ≥ 100 μM. This prediction thus provides a quantitative benchmark for the development of experimental sensors and makes it possible to assess whether a particular sensor is fast enough to accurately represent the rapid transient states of neurotransmitters.

However, determining such kinetic constants is challenging, as it, for example, requires single‐molecule measurements with high signal‐to‐noise ratios at sub‐second temporal resolution. In a recent publication, the rate constants of catecholamine ssDNA‐SWCNT sensors were measured by analyzing the fluorescence traces of single‐molecule experiments (Gretz et al. 2025). It was shown that different surface chemistries result in sensors with different rate constants for different catecholamines, and that this can be exploited to increase selectivity under non‐equilibrium conditions. As the experimentally determined rate constants differ from theoretically optimal values, kinetic models are required to reconstruct the extracellular catecholamine concentrations from the measured fluorescence signals. An open‐source simulation framework (Gretz et al. 2026) was recently introduced that enables both forward simulations of neurotransmitter release and sensor responses, as well as the solution of the inverse problem by comparing simulated and experimental image sequences to identify the most likely scenario of neurotransmitter release (Gretz et al. 2026).

Full kinetic investigations of genetically encoded sensors, including concentration dependencies, have rarely been performed, so far, but can be determined, for example, by fast‐perfusion patch clamp fluorometry measurements, as recently shown for a serotonin sensor (Kubitschke et al. 2022). The utility of profound kinetic data was recently demonstrated by the ability to reconstruct the spatiotemporal concentration of cellular signals under investigation using a deep‐neural‐network prior that was tested on several GCaMP calcium sensors and revealed distinct spatiotemporal events in the calcium distribution of single neurons (Pham et al. 2024). Therefore, there are now kinetic models and simulations available but they are just emerging as tools to interpret experimental data.

### 4.4 Dynamic Range and Concentrations

The sensor's binding properties not only set the temporal resolution but also define the concentration range over which signals can be accurately measured. Catecholamine concentrations span several orders of magnitude, from nanomolar baseline levels to millimolar levels during vesicular release, meaning that no single sensor can reliably cover the entire spectrum. As mentioned earlier, variations in the ssDNA sequences can influence the kinetics of SWCNT‐based sensors and may be tailored to specific concentration ranges/kinetic, but identifying an optimal sensor remains a time‐consuming process. This problem also applies to genetically encoded sensors. An elegant solution to this problem is provided by Labouesse et al., who used the DA receptor dLight1.3b to measure DA concentration changes in the micromolar range and then added DETQ, a known positive allosteric modulator of the underlying human D1 receptor scaffold, to increase the sensor affinity by eightfold, shifting the detection into the hundreds of nanomolar range (Labouesse et al. 2024). Such on‐demand tuning of sensor affinity could be particularly useful for disentangling diverse DA release dynamics in vivo. However, caution must be exercised when interpreting absolute catecholamine concentrations, as the measured fluorescence can be influenced by many factors. As discussed in the previous section, finite association and dissociation kinetics can lead to delayed and prolonged sensor occupancy, while high concentrations of high‐affinity sensors can additionally lead to buffering effects, which distort the observed dynamics (see e.g., (Armbruster et al. 2020)). Accurate quantification therefore requires careful calibration. Although calibration can be performed in well‐defined model systems, transferring these calibrations to in vivo experiments remains challenging because sensor responses depend on local pH, ionic strength, temperature and the biological microenvironment. Moreover, these parameters often vary simultaneously and cannot be independently controlled under physiological conditions. Depending on the sensor platform, additional factors such as expression level, membrane localization, and cellular physiology (genetically encoded sensors) or sensor concentration, the local corona phase, biomolecular adsorption, and the surrounding tissue environment (SWCNT‐based sensors) further influence the sensor response. Consequently, sensors should ideally be calibrated individually for their intended application under conditions that closely resemble the biological system of interest, with quantitative estimates expected to become more reliable as calibration conditions approach the experimental environment.

### 4.5 Imaging

From a practical perspective, genetically encoded sensors offer the advantage of being compatible with standard optical imaging setups, requiring no specialized NIR equipment beyond conventional fluorescence microscopes or fiber photometry setups.

In contrast, SWCNT‐based sensors typically demand more specialized instrumentation, including lasers and indium gallium arsenide‐based cameras, as well as expertise in sensor synthesis and surface functionalization. Recent advances, however, have begun to mitigate these limitations. For example, the use of (6,4)‐SWCNTs has enabled high‐sensitivity detection using standard microscope equipment (Ackermann, Stegemann, et al. 2023), while integration into cell culture labware and the ability to sterilize and store (Ackermann, Reger, et al. 2023) facilitates broader accessibility of these nanosensors.

Important biological insights might also come by further multiplexing, for example, analyte detection with more than two separate fluorescence emission lines. Here, SWCNTs are particularly versatile due to their different chiralities, which exhibit different fluorescence wavelengths. Their narrow emission bands (~20 nm) result in minimal spectral overlap, allowing up to 17 chiralities to be imaged simultaneously (Roxbury et al. 2015). However, studies simultaneously detecting multiple chiralities remain relatively rare, largely because of the additional effort required for chirality separation (Nißler et al. 2022). For genetically encoded sensors, the toolbox has recently been expanded beyond the red and green neuromodulator sensors to include the far‐red DA sensor HaloDA1.0, which enables parallel imaging of extracellular DA alongside acetylcholine concentrations and intracellular cAMP levels with minimal cross‐talk (Zheng et al. 2025). Owing to the broader excitation and emission spectra of fluorescent proteins, genetically encoded sensors generally offer a lower multiplexing level than SWCNTs due to increased spectral overlap. However, a higher degree of multiplexing generally comes at the expense of temporal resolution, since multiple spectral channels must be acquired. A recently developed hyperspectral imaging approach has shown that spectral information can instead be reconstructed from just three acquired images, which is particularly advantageous at high degrees of multiplexing (Stegemann et al. 2025). Furthermore, more robust imaging strategies offer additional opportunities to enhance the performance of catecholamine sensors. For example, stable, NIR‐emitting Egyptian blue nanosheets combined with SWCNT sensors have been used for ratiometric imaging of DA release, demonstrating increased robustness against mechanical disturbances (Hill et al. 2024). Similarly, NIR fluorescence lifetime imaging (FLIM) of SWCNT‐based sensors has been shown to provide readouts largely independent of sensor concentration or excitation intensity (Sistemich et al. 2023). When applied with confocal 3D imaging, this approach enables spatial mapping of extracellular DA release and more quantitative assessment of neurotransmitter dynamics. For the same reason, genetically encoded sensors with lifetime changes are highly desirable and first FLIM‐suitable variants have been reported, including dLight3.8 (Tian et al. 2025; Lodder et al. 2025; Kubitschke et al. 2024).

Overall, the full potential of these promising sensors has not yet been fully exploited, and further advances in sensor design, combined with innovative imaging strategies and kinetic data analysis could enable even more precise quantitative measurements of catecholamines in complex biological systems. In particular, the integration of multiplexed sensing with kinetic modeling may help disentangle true neurotransmitter concentration dynamics from sensor response kinetics and thereby resolve neuromodulator signaling across spatial and temporal scales. Achieving this goal will require close collaborations between synthetic chemists, protein engineers, biophysicists, neuroscientists and computational scientists. Such interdisciplinary efforts are likely to further expand the capabilities of optical catecholamine sensors and deepen our understanding of neuromodulatory signaling in the brain and other organ systems.

## Author Contributions

**Andreas Reiner:** conceptualization, writing – original draft, writing – review and editing, funding acquisition. **Sebastian Kruss:** writing – review and editing, funding acquisition, conceptualization. **Julia Ackermann:** conceptualization, writing – original draft, writing – review and editing.

## Funding

We thank the DFG for funding (Kr 4242 7/1, 8/1). This work was supported by the Deutsche Forschungsgemeinschaft (DFG, German Research Foundation) under Germany's Excellence Strategy—EXC 2033‐390677874‐RESOLV. Additionally, this work was supported by the “Center for Solvation Science ZEMOS”, which was funded by the German Federal Ministry of Education and Research (BMBF) and the Ministry of Culture and Research of North Rhine‐Westphalia. This work was funded by the German Federal Ministry of Education and Research (BMBF, SmartSlides, 13N16847). Work in the lab of A.R. is funded by DFG RTG 2862/1, “Monoaminergic Neuronal Networks & Disease”.

## Conflicts of Interest

The authors declare no conflicts of interest.

## Data Availability

The authors have nothing to report.
